# Gastrointestinal dysfunction in aneurysmal subarachnoid hemorrhage: prevalence, clinical correlates, and prognostic implications from a 15-year ICU cohort study

**DOI:** 10.3389/fneur.2025.1694188

**Published:** 2025-12-17

**Authors:** Tongjuan Zou, Hao He, You Wu, Xiaoqi Xie, Wanhong Yin

**Affiliations:** 1 Department of Critical Care Medicine, West China School of Medicine, West China Hospital, Sichuan University, Chengdu, Sichuan, China; 2 Visualized Diagnostics and Therapeutics & Artificial Intelligence Laboratory (VDT-AI Lab), West China Hospital, Sichuan University, Chengdu, China

**Keywords:** aneurysmal subarachnoid hemorrhage, gastrointestinal dysfunction, Hunt-Hess score, autonomic dysregulation, neurocritical care

## Abstract

**Background:**

Gastrointestinal dysfunction (GID) is increasingly recognized in neurocritical care, but disease-specific epidemiology, associated clinical factors, and outcomes in aneurysmal subarachnoid hemorrhage (aSAH) remain insufficiently characterized. We aimed to quantify the prevalence of GID in patients with aSAH, identify its clinical associations, and evaluate its prognostic implications.

**Methods:**

We conducted a 15-year retrospective cohort study involving consecutive adults with aSAH who were admitted to the neurological intensive care unit (NICU) at West China Hospital (24 October 2009–29 June 2024). GID was defined pragmatically as the presence of the following symptoms/signs: gastric residual volume [GRV] ≥ 500 mL on any calendar day after enteral nutrition initiation, gastrointestinal bleeding, or Bristol-defined diarrhea. GID occurrence was modeled using Fine-Gray competing-risk analysis (with in-hospital death as the competing event). In-hospital mortality was analyzed using multivariable logistic regression. Thirty-day survival was described by Kaplan–Meier (KM) curves.

**Results:**

Among the 994 patients with aSAH, GID occurred in 44.8% (445/994). Compared to non-GID patients, those with GID had higher admission heart rates and temperature levels, along with a greater proportion having a Hunt-Hess (HH) score ≥4 (43% vs. 20%, *p* < 0.001). Patients with GID had significantly longer ICU (18.5 ± 14.8 vs. 6.2 ± 5.7 days) and hospital stays (26.5 ± 20.5 vs. 12.7 ± 8.4 days) and higher in-hospital mortality (37% vs. 22%, *p* < 0.001). The GID group also had higher levels of N-terminal pro-B-type natriuretic peptide (NT-proBNP) (1576.76 ± 3237.84 vs. 1251.52 ± 2673.15, *p* = 0.023), C-reactive protein (CRP) (62.64 ± 69.30 vs. 39.84 ± 56.95, *p* < 0.001), interleukin-6 (IL-6) (136.03 ± 355.40 vs. 77.64 ± 182.79, *p* < 0.001), and procalcitonin (PCT) (1.07 ± 5.71 vs. 0.56 ± 2.88, *p* < 0.001). In the multivariable Fine-Gray competing-risk analysis, nasojejunal tube use, arrhythmia, target temperature management, HH ≥ 4, and GI drug exposure were associated with a higher subdistribution hazard of GID. KM curves showed lower unadjusted 30-day survival in the GID group (log-rank *p* < 0.0001). GID was not independently associated with in-hospital mortality in multivariable analyses.

**Conclusion:**

In aSAH, GID is common and correlates with neurological severity, autonomic dysregulation, systemic inflammation, and resource use. Although GID is not independently associated with mortality after adjustment, it identifies a high-risk subgroup and supports early, structured gastrointestinal supportive strategies in neurocritical care.

## Introduction

Aneurysmal subarachnoid hemorrhage (aSAH) is a catastrophic hemorrhagic stroke with a major global public health impact (1, 2). The Global Burden of Disease (GBD) 2021 analysis reported an age-standardized incidence of subarachnoid hemorrhage of 8.33 per 100,000 person-years (95% UI, 7.34–9.48) and an age-standardized mortality rate of 4.23 per 100,000 person-years, accounting for significant years of life lost and disability-adjusted life years worldwide (1, 3). Although the mortality rate has declined modestly in some regions with advances in neurocritical care, early mortality rates, particularly prehospital mortality, remains high, with estimates ranging from 22 to 26% (4–6).

Non-traumatic SAH is most commonly caused by rupture of an intracranial aneurysm, accounting for approximately 85% of cases (7). Established risk factors for the condition include advanced age, chronic hypertension, tobacco smoking, excessive alcohol use, and genetic predispositions such as autosomal dominant polycystic kidney disease (1, 8). Regional disparities in incidence and outcomes likely reflect variations in risk factor prevalence, access to specialized neurocritical care, and implementation of guideline-based management (3, 7).

Autonomic nervous system (ANS) dysregulation is a frequent systemic complication of aSAH, with reported rates of 20–40% and exhibits a higher prevalence in patients with greater neurological severity, as indicated by a Hunt-Hess score ≥4 (9, 10). Dysautonomia after aSAH may manifest as cardiovascular instability, arrhythmias, blood pressure lability, thermoregulatory disturbances, and gastrointestinal dysfunction (GID) (11), mediated by hypothalamic injury, catecholamine surge, and disruption of brainstem autonomic centers (9, 11, 12).

In neurocritical care, GID is increasingly recognized as a clinically relevant complication that affects feeding tolerance, infection risk, and recovery (13, 14). In aSAH, the proposed mechanisms of GID include sympathetic overactivation, systemic inflammation, blood-brain barrier disruption, altered gut permeability, and dysbiosis along the brain-gut-microbiota axis (10, 15). These pathophysiological processes may lead to delayed gastric emptying, intestinal dysmotility, feeding intolerance, and malabsorption, with potential implications for outcomes and resource utilization (13, 15).

For this study, GID was defined as any of the following: gastrointestinal symptoms (such as vomiting, abdominal distension, or abdominal pain), gastric residual volume (GRV) of ≥ 500 mL within any calendar day (00:00–24:00) after initiation of enteral nutrition (EN) during the ICU stay, gastrointestinal bleeding, or diarrhea according to the Bristol Stool Form Scale. Although GID has been described in general ICU cohorts, disease-specific epidemiology, clinical correlates, and outcomes of GID in aSAH remain incompletely characterized (13, 16). Large, adequately powered studies that quantify the GID burden in aSAH and examine its relationship to neurological severity and healthcare use are scarce (17). Accordingly, we aimed to determine the prevalence of GID in aSAH, describe its clinical associations, and evaluate its prognostic implications in a 15-year ICU cohort.

## Materials and methods

### Ethics approval and consent to participate

This study was approved by the Institutional Review Board of West China Hospital (Approval No. 20221424). The requirement for informed consent was waived because of the minimal-risk, retrospective design of the study.

### Study design and setting

#### Selection and description of participants

We conducted a retrospective cohort study (October 2009–June 2024) at the neurological intensive care unit (NICU) of West China Hospital, Sichuan University. The inclusion criterion was adults (≥18 years) with imaging-confirmed aSAH. The exclusion criteria were pregnancy and missing any essential field required for analysis, defined *a priori* as (i) neurological grade (Hunt-Hess score), (ii) gastrointestinal documentation sufficient to adjudicate the GID status, or (iii) discharge vital status (alive/dead).

### Data collection

Data were retrospectively collected from the electronic medical records system of the ICU during the study period. Baseline variables included demographics, vital signs at hospital admission (temperature, heart rate, respiratory rate, systolic and diastolic blood pressure, and level of consciousness), laboratory results (e.g., C-reactive protein [CRP], interleukin-6 [IL-6], procalcitonin [PCT], N-terminal pro-B-type natriuretic peptide [NT-proBNP]), and treatment. Neurological severity was summarized using the Hunt-Hess score, and functional outcome at hospital discharge was summarized as Glasgow Outcome Scale (GOS) ≤ 3 (unfavorable) vs. ≥4 (favorable). Surgery was defined as any neurosurgical intervention, including aneurysm repair by microsurgical clipping or endovascular coiling, decompressive craniectomy, and external ventricular drainage. We also recorded nasojejunal tube (NJT) use, time to enteral nutrition (EN), gastrointestinal (GI) drug exposure (e.g., prokinetics), and target temperature management (TTM). TTM exposure was abstracted from ICU orders and nursing temperature management records and coded as yes/no if administered on the day of GID adjudication during the ICU stay. The primary discharge diagnosis was obtained from the discharge records. Gastrointestinal symptoms and gastric residual volume (GRV) were extracted from the nursing and progress notes. Clinical outcomes included ICU length of stay, total hospital length of stay, and in-hospital mortality.

### Definitions of gastrointestinal dysfunction

GID was diagnosed when at least one of the following criteria was present: (1) gastrointestinal symptoms or signs (vomiting, abdominal distension, or abdominal pain); (2) GRV ≥ 500 mL within any calendar day (00:00–24:00) after initiation of enteral nutrition (EN) during the ICU stay (13, 18–20); (3) gastrointestinal bleeding (GIB); or (4) diarrhea classified by the Bristol Stool Form Scale (21).

### Statistical analysis

Statistical analyses were performed using the Statistical Package for the Social Sciences (SPSS) version 27.0 and R version 4.4.3. Continuous variables were presented as mean ± standard deviation or median (interquartile range), as appropriate, and categorical variables as counts (percentages). Group comparisons were performed using *t*-tests or Wilcoxon rank-sum tests for continuous variables and χ^2^ or Fisher’s exact tests for categorical variables.

Covariates were selected *a priori* based on clinical relevance and the literature. GID occurrence was analyzed using a Fine-Gray competing-risk model (in-hospital death as the competing event), with subdistribution hazard ratios (sHRs) and 95% confidence intervals (CIs) reported. Hospital mortality was analyzed using multivariate logistic regression, with odds ratios (ORs) and 95% CIs reported. Thirty-day survival was described using Kaplan–Meier curves and log-rank tests (e.g., by GID status and Hunt-Hess score category [≥4 vs. ≤3]). Two-sided *p*-values of <0.05 were considered statistically significant.

## Results

### Study population and flow

**Figure 1:**
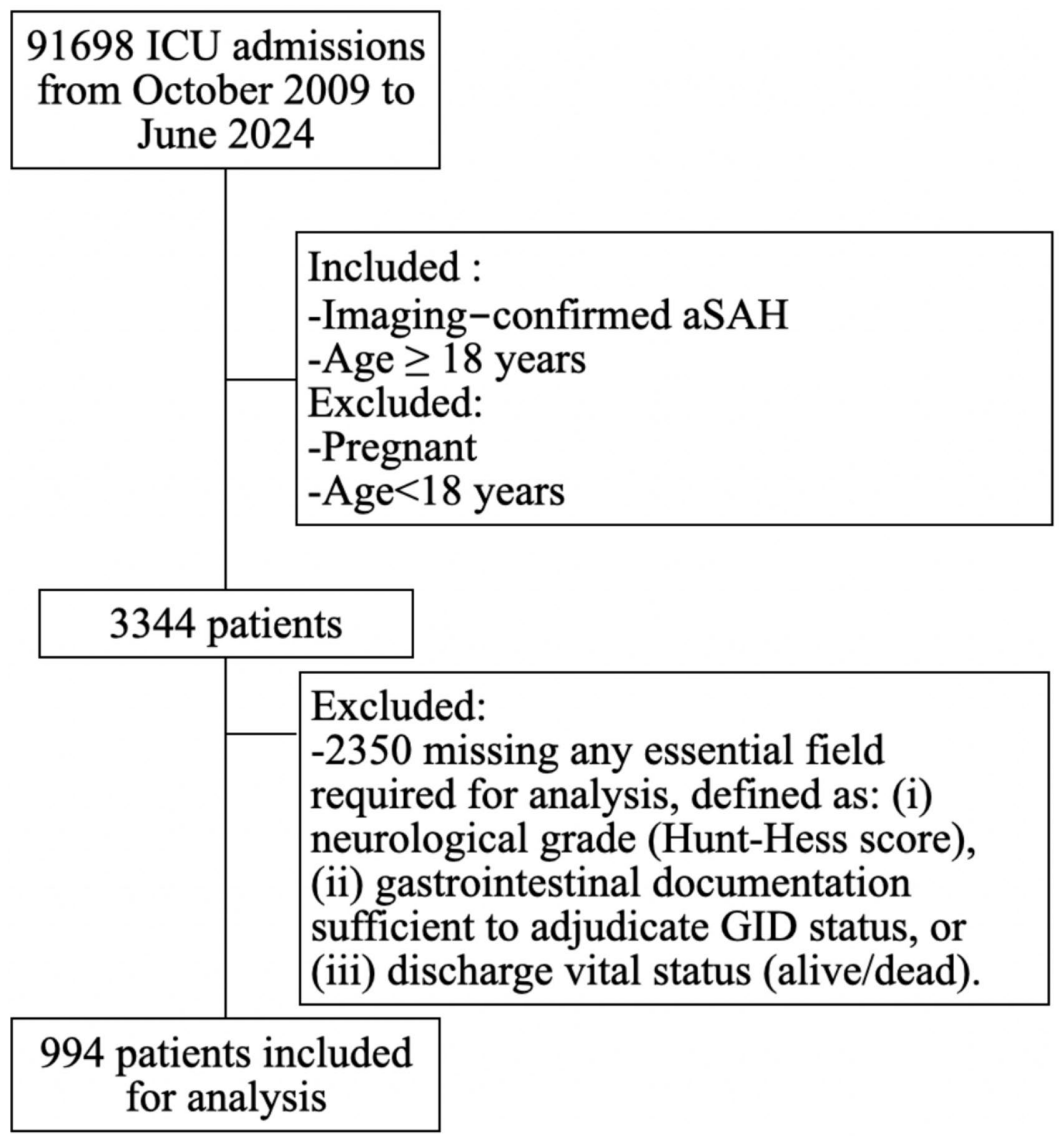
Study flow diagram. ICU, intensive care unit; aSAH, aneurysmal subarachnoid hemorrhage. From October 2009 to June 2024, 3,344 patients with aneurysmal subarachnoid hemorrhage (aSAH) were screened. After excluding 2,350 patients with incomplete data, 994 patients were included in the final analysis. aSAH, aneurysmal subarachnoid hemorrhage.

Between October 2009 and June 2024, 3,344 patients with a confirmed diagnosis of aSAH were screened. Of these, 994 patients were included in the final analysis, and 2,350 were excluded for incomplete neurological, gastrointestinal, or outcome data (Figure 1).

### Baseline characteristics at admission

The mean age was 57.56 ± 12.49 years, while the male-to-female ratio was 0.64:1. Upon hospital admission, the mean temperature was 36.82 ± 0.58 °C, heart rate (HR) was 82.28 ± 18.34 beats/min, systolic blood pressure (SBP) was 145.71 ± 23.50 mmHg, diastolic blood pressure (DBP) was 85.67 ± 14.98 mmHg, and body mass index (BMI) was 23.98 ± 3.58 kg/m^2^. A Hunt-Hess (HH) score of ≥4 was present in 30% (301/994) of the patients. The mean hospital length of stay was 18.86 ± 16.59 days, the ICU length of stay was 11.68 ± 12.38 days, and the in-hospital mortality rate was 29.0% (285/994) (Table 1).

**Table 1:** Baseline characteristics at admission and clinical outcomes stratified by GID status and Hunt–Hess score.

Variables	All patients (*n* = 994)	GID (*n* = 445)	Non-GID (*n* = 549)	*p*	HH score≥4 (*n* = 301)	HH score≤3 (*n* = 693)	*p*
Age, year	57.56 ± 12.49	60.0 ± 12.9	56.0 ± 11.8	<0.001	59.02 ± 12.15	56.93 ± 12.59	0.015
Male, *n* (%)	390 (39.2)	90 (20.22)	300 (54.6)	<0.001	136 (45.1)	254 (36.65)	0.011
Weight, kg	62.69 ± 11.56	63.26 ± 11.53	62.22 ± 11.57	0.12	63.97 ± 12.54	62.13 ± 11.07	0.048
Height, cm	161.40 ± 8.16	161.29 ± 8.24	161.49 ± 8.11	0.7	162.50 ± 8.33	160.92 ± 8.05	0.005
BMI, kg/m^2^	23.98 ± 3.58	24.24 ± 3.58	23.78 ± 3.57	0.021	24.14 ± 3.95	23.91 ± 3.41	0.5
Temperature, °C	36.82 ± 0.58	36.90 ± 0.66	36.76 ± 0.50	0.006	37.03 ± 0.78	36.74 ± 0.45	<0.001
HR, beats/min	82.28 ± 18.34	85.65 ± 19.40	79.54 ± 16.96	<0.001	89.14 ± 21.98	79.30 ± 15.61	<0.001
RR, breaths/min	19.52 ± 2.44	19.68 ± 2.86	19.39 ± 2.03	0.085	19.28 ± 3.82	19.63 ± 1.48	0.055
SBP, mmHg	145.71 ± 23.50	147.04 ± 25.66	144.63 ± 21.56	0.2	146.82 ± 26.38	145.23 ± 22.14	0.6
DBP, mmHg	85.67 ± 14.98	85.35 ± 15.26	85.93 ± 14.76	0.4	86.63 ± 17.01	85.26 ± 14.00	0.4
HH score				<0.001			<0.001
HH score ≤3, *n* (%)	693 (70)	255 (57)	438 (80)		0	693 (100)	
HH score ≥4, *n* (%)	301 (30.0)	190 (43.0)	111 (20.0)		301 (100)	0	
Underlying disease							
Hypertension, *n* (%)	480 (48)	231 (52)	249 (45)	0.04	161 (53)	319 (46)	0.031
Diabetes, *n* (%)	160 (16)	84 (19)	76 (14)	0.032	54 (18)	106 (15)	0.3
CHD, *n* (%)	60 (6.0)	27 (6.1)	33 (6.0)	0.9	20 (6.6)	40 (5.8)	0.6
Cerebral Infarction, *n* (%)	37 (3.7)	17 (3.8)	20 (3.6)	0.9	12 (4.0)	25 (3.6)	0.8
Intervention							
Operation, *n* (%)	867 (87)	374 (84)	493 (90)	0.007	226 (75.0)	641 (92)	<0.001
TTM, *n* (%)	210 (21)	145 (33)	65 (12)	<0.001	103 (34.0)	107 (15.0)	<0.001
GI-drug use, *n* (%)	609 (61)	298 (67)	311 (57)	<0.001	175 (58.0)	434 (63)	0.2
Days from ICU admission to initiation of EN, d	3.01 (3.24)	3.99 (3.43)	2.20 (2.84)	<0.001	2.94 ± 2.94	3.03 ± 3.36	0.4
NJT, *n* (%)	222 (22)	181 (41)	41 (7.5)	<0.001	113 (38.0)	109 (16.0)	<0.001
Antibiotics, *n* (%)	783 (79)	345 (78)	438 (80)	0.4	231 (77.0)	552 (80.0)	0.3
Outcome							
Hospital length of stay, d	18.86 ± 16.59	26.51 ± 20.54	12.66 ± 8.42	<0.001	23.42 ± 20.53	16.88 ± 14.11	<0.001
ICU length of stay, d	11.68 ± 12.38	18.50 ± 14.78	6.16 ± 5.68	<0.001	17.13 ± 15.92	9.32 ± 9.56	<0.001
Hospital mortality, *n* (%)	285 (29.0)	163 (37.0)	122 (22.0)	<0.001	148 (49.0)	137 (20.0)	<0.001
GID, *n* (%)	445 (44.76)	445 (100)	0	<0.001	190 (63.0)	255 (37.0)	<0.001
Functional outcome at discharge							
GOS				<0.001			<0.001
GOS ≤ 3, *n* (%)	537 (54)	341 (62)	196 (44)		454 (66)	83 (28)	
GOS ≥ 4, *n* (%)	457 (46)	208 (38)	249 (56)		239 (34)	218 (72)	

Values are presented as mean ± SD or *n* (%), as appropriate. *P*-values are used to compare the indicated subgroups. Operation is a composite variable that includes clipping/coiling, decompressive craniectomy, and EVD (coded as yes/no). BMI, body mass index; CHD, coronary heart disease; CKD, chronic kidney disease; DBP, diastolic blood pressure; GI, gastrointestinal; GID, gastrointestinal dysfunction; GOS, Glasgow Outcome Scale; HH score, Hunt–Hess score; HR, heart rate; ICU, intensive care unit; NJT, nasojejunal tube; RR, respiratory rate; SBP, systolic blood pressure.

### Comparison by GID status

Among the 994 patients, 445 (44.77%) developed GID. Compared with the non-GID group, patients with GID were older (60.0 ± 12.9 vs. 56.0 ± 11.8, *p* < 0.001), had higher temperature levels at admission (36.90 ± 0.66 vs. 36.76 ± 0.50, *p* = 0.006), higher BMI (24.24 ± 3.58 vs. 23.78 ± 3.57, *p* = 0.021), and faster HR (85.65 ± 19.40 vs. 79.54 ± 16.96, *p* < 0.001). An HH score of ≥ 4 was more frequent in the GID group (43.0% [190/445] vs. 20% [111/549], *p* < 0.001). Patients with GID had longer hospital stays (26.51 ± 20.54 vs. 12.66 ± 8.42, *p* < 0.001) and ICU stays (18.50 ± 14.78 vs. 6.16 ± 5.68, *p* < 0.001) and higher in-hospital mortality (37.0% vs. 22.0%, *p* < 0.001). A GOS score of ≤ 3 was more frequent in the GID group (62.0% vs. 44%, *p* < 0.001) (Table 1).

### Comparison by neurological severity (HH score)

When stratified by neurological severity, patients with an HH score ≥4 were older (59.02 ± 12.15 vs. 56.93 ± 12.59, *p* = 0.015), had higher admission temperatures (37.03 ± 0.78 vs. 36.74 ± 0.45, *p* < 0.001), and had faster HR (89.14 ± 21.98 vs. 79.03 ± 15.61, *p* < 0.001) than those with an HH score ≤ 3. Patients with an HH score ≥4 also had longer hospital stays (23.42 ± 20.53 vs. 16.88 ± 14.11 days; *p* < 0.001) and ICU stays (17.13 ± 15.92 vs. 9.32 ± 9.56 days; *p* < 0.001) as well as higher rates of GID (63% vs. 37%; *p* < 0.001) and in-hospital mortality (49% vs. 20%; *p* < 0.001). A GOS score ≤ 3 was more frequent among patients with an HH score ≥4 (66.0% vs. 28%, *p* < 0.001) (Table 1).

### Laboratory findings by GID status and Hunt-Hess score

The admission laboratory tests are summarized in Table 2. Compared to patients without GID, those with GID had higher leukocyte counts, renal indices, liver enzymes, myocardial injury markers, inflammatory biomarkers, and coagulation markers, and lower platelet counts, hemoglobin levels, and albumin levels (Table 2). Similarly, patients with a Hunt-Hess score ≥4 had more abnormal hematological, renal, hepatic, cardiac, and inflammatory markers than those with scores ≤3 (Table 2).

**Table 2:** Admission laboratory tests stratified by GID status and Hunt–Hess score.

Variables	All patients (*n* = 994)	GID (*n* = 445)	Non-GID (*n* = 549)	*p*	HH score≥4 (*n* = 301)	HH score≤3 (*n* = 693)	*p*
RBC, ×10^12^/L	3.84 (0.77)	3.81 (0.84)	3.87 (0.70)	0.1	3.74 (0.79)	3.88 (0.75)	0.005
WBC, ×10^9^/L	10.87 (4.35)	11.20 (4.46)	10.59 (4.24)	0.018	12.66 (5.00)	10.09 (3.78)	<0.001
PLT, ×10^9^/L	165.54 (69.31)	160.27 (72.37)	169.81 (66.50)	0.002	159.17 (76.47)	168.31 (65.83)	0.003
Hgb, g/L	115.53 (22.88)	114.25 (24.52)	116.56 (21.42)	0.037	113.08 (24.53)	116.59 (22.05)	0.004
Lym count, ×10^9^/L	0.96 (0.52)	0.94 (0.51)	0.97 (0.52)	0.2	0.86 (0.42)	1.00 (0.55)	0.002
Cr, μmol/L	72.46 (74.89)	75.07 (79.08)	70.34 (71.32)	0.018	82.14 (97.73)	68.25 (62.04)	<0.001
BUN, mmol/L	4.97 (2.77)	5.22 (3.02)	4.76 (2.54)	0.025	5.41 (3.20)	4.77 (2.54)	0.021
TP, g/L	60.62 (8.72)	60.15 (9.13)	61.00 (8.36)	0.1	59.47 (9.57)	61.12 (8.28)	0.004
ALB, g/L	35.46 (7.02)	34.73 (7.29)	36.06 (6.74)	0.002	34.65 (7.47)	35.82 (6.79)	0.004
TBIL, μmol/L	12.60 (7.02)	12.91 (7.27)	12.34 (6.80)	0.2	13.60 (7.24)	12.16 (6.88)	<0.001
DBIL, μmol/L	4.31 (2.96)	4.42 (3.16)	4.22 (2.78)	0.6	4.67 (3.00)	4.16 (2.93)	<0.001
IBIL, μmol/L	8.28 (4.87)	8.49 (4.93)	8.12 (4.81)	0.15	8.93 (5.27)	8.00 (4.66)	0.009
ALT, IU/L	23.72 (26.67)	25.21 (28.93)	22.51 (24.65)	0.027	25.93 (25.32)	22.76 (27.19)	<0.001
AST, IU/L	26.90 (25.92)	27.97 (25.15)	26.04 (26.52)	0.005	32.92 (26.83)	24.29 (25.09)	<0.001
TnT, ng/L	54.99 (139.21)	67.18 (160.44)	45.10 (118.50)	<0.001	107.16 (200.53)	32.33 (93.15)	<0.001
CK, U/L	204.81 (285.13)	218.87 (293.83)	193.42 (277.63)	0.067	278.43 (389.67)	172.84 (217.84)	<0.001
Myo, ng/mL	187.85 (242.77)	217.48 (295.89)	163.83 (185.94)	0.13	265.05 (354.99)	154.32 (161.94)	<0.001
CK-MB, U/L	4.01 (4.44)	4.23 (4.58)	3.82 (4.33)	0.2	5.16 (5.31)	3.51 (3.91)	<0.001
D-Dimer, mg/L	5.41 (6.11)	6.24 (6.83)	4.73 (5.36)	<0.001	6.80 (6.83)	4.80 (5.66)	<0.001
PT, s	11.52 (1.50)	11.67 (1.63)	11.40 (1.37)	0.007	11.87 (1.76)	11.37 (1.34)	<0.001
APTT, s	29.03 (9.16)	30.00 (11.92)	28.24 (5.95)	0.071	30.49 (12.70)	28.39 (7.00)	0.008
NT-proBNP, pg./mL	1,397.13 (2,942.31)	1,576.76 (3,237.84)	1,251.52 (2,673.15)	0.023	1,847.44 (3,226.58)	1,201.54 (2,789.79)	<0.001
CRP, mg/L	50.05 (63.76)	62.64 (69.30)	39.84 (56.95)	<0.001	74.65 (70.03)	39.36 (57.71)	<0.001
IL-6, pg./mL	103.78 (275.24)	136.03 (355.40)	77.64 (182.79)	<0.001	135.35 (337.24)	90.06 (242.45)	<0.001
PCT, ng/mL	0.79 (4.38)	1.07 (5.71)	0.56 (2.88)	<0.001	1.72 (7.32)	0.39 (1.94)	<0.001

Values are mean ± SD. Laboratory units follow institutional standards. aSAH, aneurysmal subarachnoid hemorrhage; ALB, albumin; ALT, alanine aminotransferase; APTT, activated partial thromboplastin time; AST, aspartate aminotransferase; BUN, blood urea nitrogen; CK, creatine kinase; CK-MB, creatine kinase-MB; Cr, creatinine; CRP, C-reactive protein; DBIL, direct bilirubin; GID, gastrointestinal dysfunction; Hgb, hemoglobin; HH score, Hunt–Hess score; IBIL, indirect bilirubin; IL-6, interleukin-6; Lym, lymphocyte count; Myo, myoglobin; NT-proBNP, N-terminal pro-B-type natriuretic peptide; PCT, procalcitonin; PLT, platelet count; PT, prothrombin time; RBC, red blood cell count; TBIL, total bilirubin; TnT, troponin T; TP, total protein; WBC, white blood cell count.

### Association between clinical indices and GID

#### Multivariable Fine-Gray competing-risk analysis of factors associated with GID

**Figure 2:**
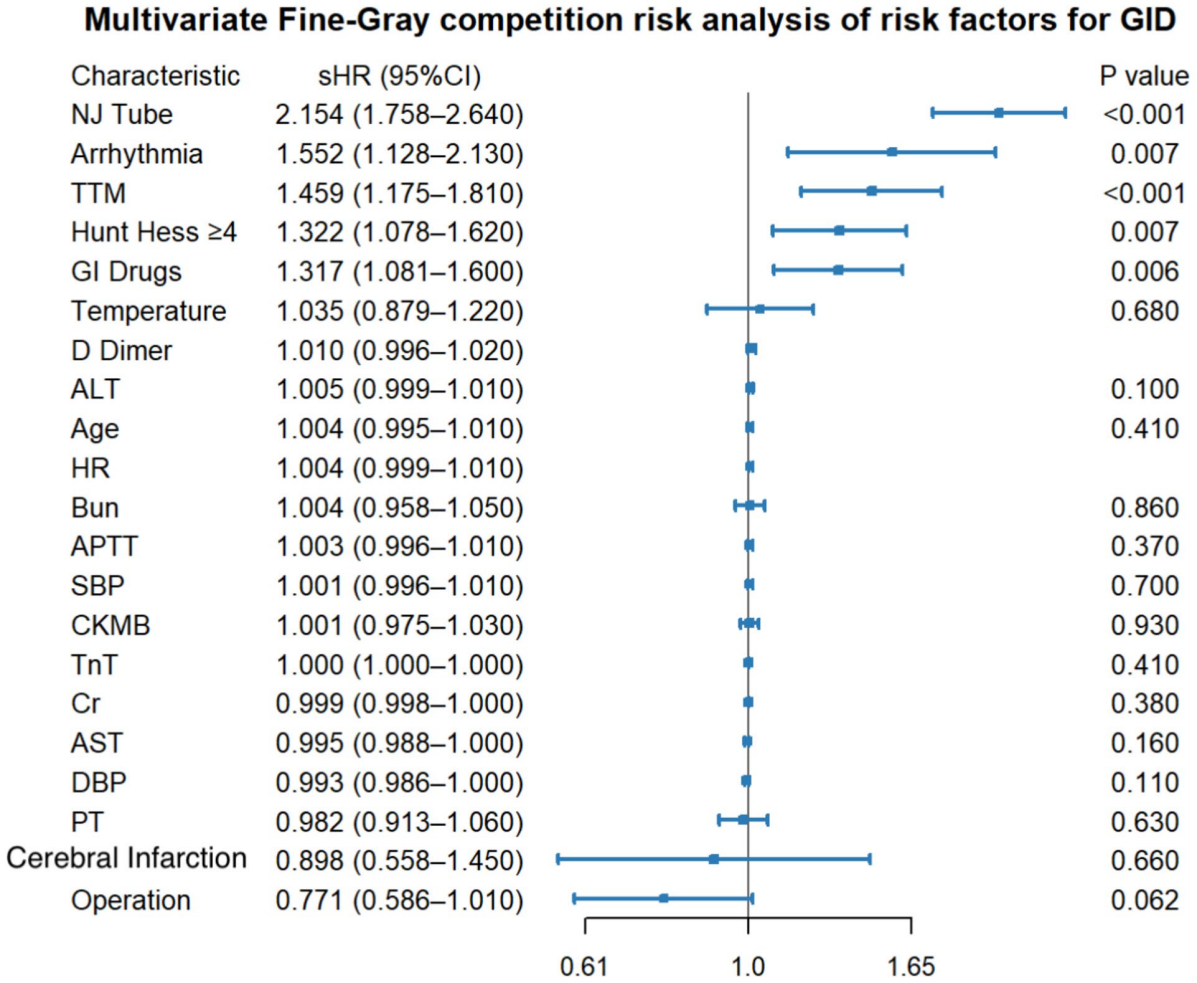
Multivariable Fine-Gray competing-risk analysis of factors associated with GID (death as the competing event). Points show sHRs with 95% CIs. Two-sided *p* < 0.05 was considered statistically significant. APTT, activated partial thromboplastin time; ALT, alanine aminotransferase; AST, aspartate aminotransferase; BUN, blood urea nitrogen; CI, confidence interval; CK-MB, creatine kinase-MB; Cr, creatinine; DBP, diastolic blood pressure; GID, gastrointestinal dysfunction; GI, gastrointestinal; NJT, nasojejunal tube; PCT, procalcitonin; PT, prothrombin time; SBP, systolic blood pressure; sHR, subdistribution hazard ratio; TnT, troponin T; TTM, target temperature management; WBC, white blood cell count.

In the multivariable Fine-Gray competing-risk model (death as the competing event), nasojejunal tube use (sHR 2.15, 95% CI 1.76–2.64), arrhythmia (sHR 1.55, 95% CI 1.13–2.13), target temperature management (sHR 1.46, 95% CI 1.18–1.81), a Hunt-Hess score ≥4 (sHR 1.32, 95% CI 1.08–1.62), and GI drug exposure (sHR 1.32, 95% CI 1.08–1.60) were associated with a higher subdistribution hazard of GID. Operation showed a non-significant trend toward a lower GID incidence (sHR 0.77, 95% CI 0.59–1.01; *p* = 0.062), while other covariates were not significant after adjustment (Figure 2).

### Association between clinical indices and hospital mortality

**Figure 3:**
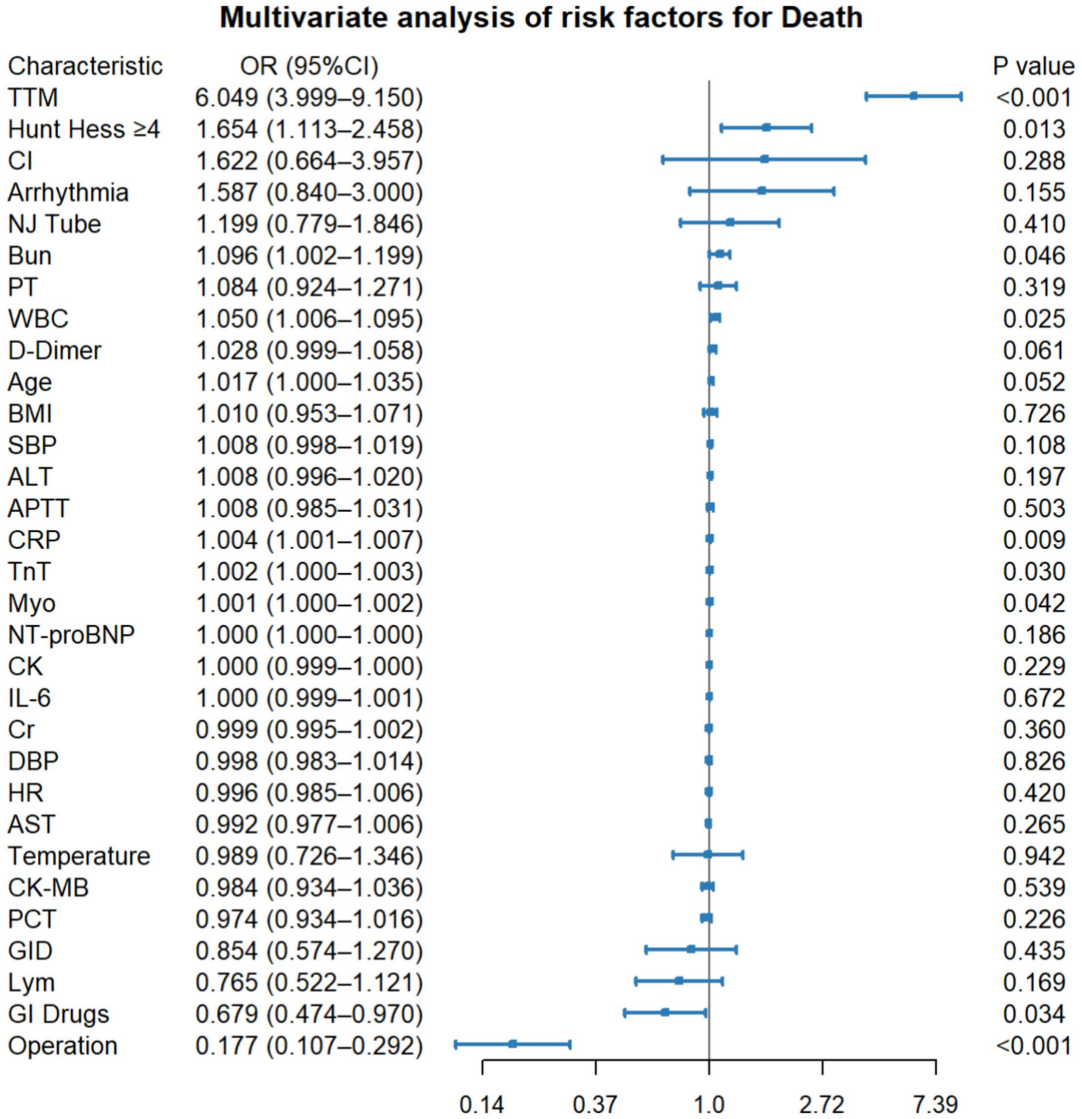
Forest plot of multivariable associations with in-hospital mortality. Odds ratios (ORs) with 95% confidence intervals (CIs) are reported. Two-sided *p* < 0.05 is considered statistically significant. APTT, activated partial thromboplastin time; ALT, alanine aminotransferase; AST, aspartate aminotransferase; BMI, body mass index; BUN, blood urea nitrogen; CI, confidence interval; CK, creatine kinase; CK-MB, creatine kinase-MB; CRP, C-reactive protein; Cr, creatinine; DBP, diastolic blood pressure; GID, gastrointestinal dysfunction; GI, gastrointestinal; HR, heart rate; IL-6, interleukin-6; Lym, lymphocyte count; Myo, myoglobin; NJT, nasojejunal tube; NT-proBNP, N-terminal pro-B-type natriuretic peptide; OR, odds ratio; PCT, procalcitonin; PT, prothrombin time; SBP, systolic blood pressure; TnT, troponin T; TTM, target temperature management; WBC, white blood cell count.

In multivariable logistic regression, TTM, HH score≥4, BUN, WBC, CRP, TnT, and Myo were independently associated with increased mortality (*p* < 0.001, 0.013, 0.046, 0.025, 0.009, 0.030, and 0.042, respectively), whereas operation and GI drug use were associated with lower mortality (*p* < 0.001 and *p* = 0.034, respectively). GID was not independently associated with in-hospital mortality in the multivariable logistic model (OR 0.854, 95% CI 0.574–1.270, *p* = 0.435) (Figure 3).

### Survival analysis

**Figure 4:**
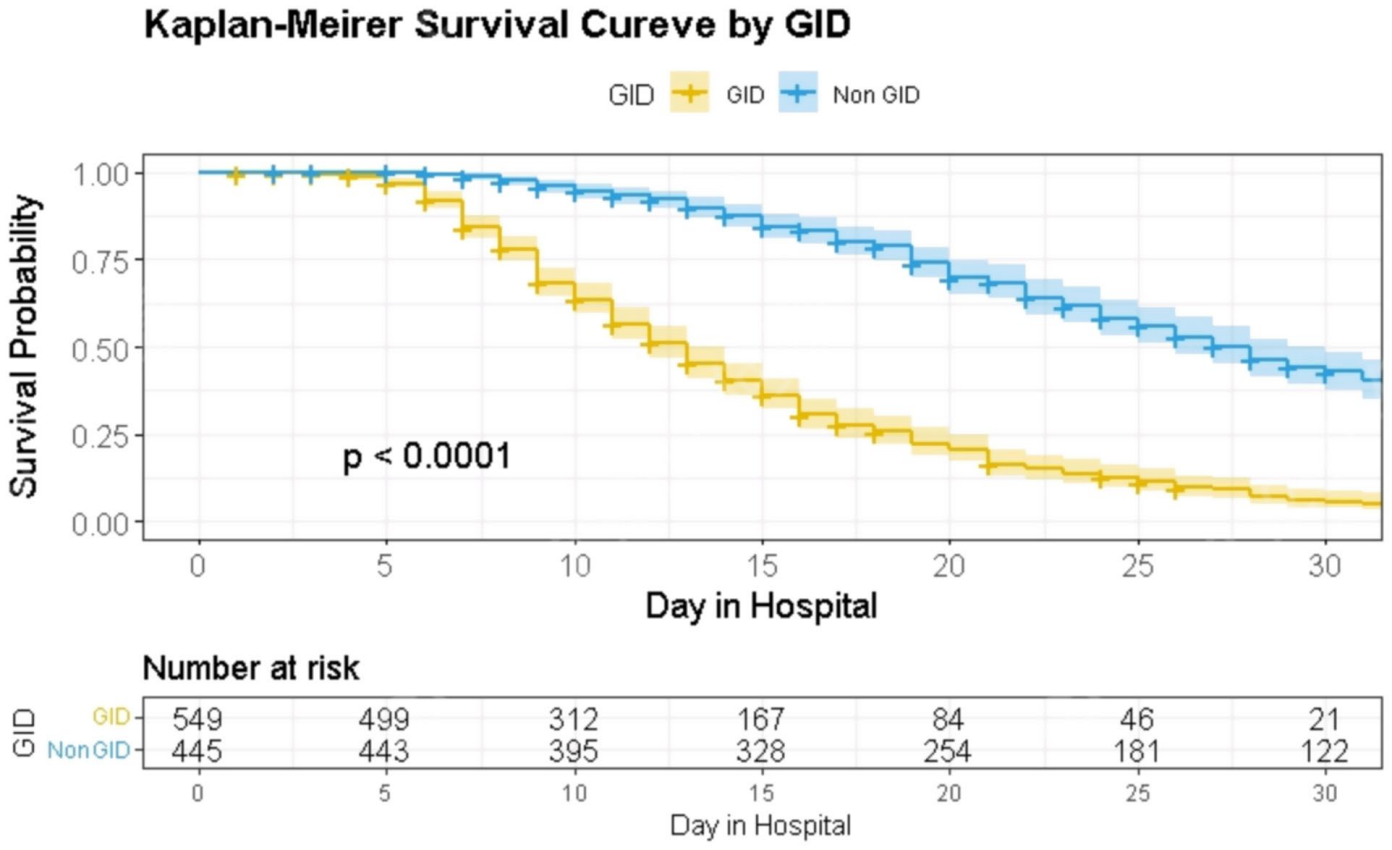
Kaplan–Meier curves for 30-day survival by GID status. Patients with GID had significantly lower survival rates (log-rank *p* < 0.0001). GID, gastrointestinal dysfunction.

Thirty-day survival differed significantly between the GID and non-GID groups; Kaplan–Meier curves showed lower unadjusted survival in patients with GID (log-rank *p* < 0.0001) (Figure 4).

## Discussion

In this 15-year ICU cohort of 994 adults with aneurysmal subarachnoid hemorrhage (aSAH), gastrointestinal dysfunction (GID) affected approximately 45% of the patients. GID was associated with greater neurological severity, longer ICU and hospital stays, and increased in-hospital mortality. Kaplan–Meier curves showed lower 30-day survival in patients with GID. In multivariable Fine-Gray competing-risk models, nasojejunal tube use, arrhythmia, target temperature management, a Hunt-Hess score ≥ 4, and GI drug exposure were associated with a higher subdistribution hazard of GID. Disease severity, as reflected by higher Hunt-Hess (HH) scores, was a major determinant of GID occurrence, with patients with an HH score of ≥ 4 exhibiting markedly higher rates of dysfunction. Patients with GID or with an HH score of ≥ 4 also had higher heart rates at admission and increased body temperature levels, suggesting a more pronounced autonomic dysregulation. Meanwhile, patients with GID or with an HH score of ≥ 4 also showed poorer hepatic, renal, and cardiac indices and more pronounced inflammatory responses, suggesting systemic responses beyond isolated gastrointestinal impairment. Collectively, these findings indicate that GID is common and clinically consequential in aSAH, indicating risk stratification and GI-focused supportive care.

The burden of GI impairment in critical illness is increasingly recognized, but disease-specific evidence in aSAH remains limited (15, 20, 22, 23). Within neurocritical care, autonomic nervous system dysfunction—including excessive sympathetic activation—together with neuroinflammation, blood-brain barrier disruption, and perturbations of the brain-gut-microbiota axis are key mechanisms mediating GI injury after acute brain injury (17, 24, 25). Emerging aSAH-specific evidence further demonstrates reduced enteral drug absorption and altered gut microbiome profiles (10, 26), reinforcing the biological plausibility of our clinical associations (17, 27, 28). The definition of gastrointestinal dysfunction (GID) in critical care lacks a unified standard. Previous studies on general ICU populations have underscored the frequency and clinical relevance of enteral feeding intolerance (EFI) and the need for harmonized criteria (e.g., GRV thresholds and symptom-based definitions). Consensus frameworks such as the Acute Gastrointestinal Injury (AGI) grading (20) and the newer Gastrointestinal Dysfunction Score (GIDS) (13) have been proposed to standardize assessment and prognostication and are undergoing validation in diverse ICU settings (13, 18, 29). Few aSAH-specific cohorts have systematically quantified the prevalence of GID and outcomes (10, 15). In this study, using a pragmatic case definition based on EHR (symptoms/signs, GRV ≥ 500 mL on any calendar day after EN initiation, GI bleeding, or Bristol-defined diarrhea), 45% of aSAH patients met the criteria for GID. GID correlated with higher neurological severity and higher crude mortality, consistent with the literature linking autonomic instability and inflammatory activation to worse trajectories after acute brain injury. Importantly, this study leverages a large, 15-year, single-center cohort with detailed clinical, physiological, and outcome data, thereby providing one of the most comprehensive characterizations of GID in aSAH to date.

From a clinical perspective, these associations have several implications. First, early screening for GID in patients with higher Hunt-Hess scores and in those receiving TTM may facilitate the timely initiation of targeted GI supportive measures (e.g., prokinetics, post-pyloric feeding, and close monitoring of gastric residuals). Second, the independent associations between inflammatory biomarkers (e.g., CRP), GID, and mortality underscore the value of integrating systemic inflammation into risk stratification. Third, the inverse associations of operation and GI drug use with mortality in adjusted models likely reflect confounding by indication and timely definitive care rather than direct protection, and therefore warrant cautious interpretation.

This study has several limitations. First, its retrospective, single-center design may limit generalizability, as management strategies and patient characteristics can differ across institutions and may have evolved over the 15-year period, potentially introducing residual confounding factors. Second, our pragmatic GID definition combined clinician-documented symptoms/signs with objective elements (GRV threshold, GI bleeding, and Bristol-defined diarrhea); such criteria are consistent with ICU practice but are vulnerable to documentation variability and GRV-monitoring heterogeneity. Third, neurological complications and imaging-based severity measures commonly used in aSAH—most notably delayed cerebral ischemia (DCI), rebleeding, hydrocephalus, and the modified Fisher (mFisher) scale—were not included in the primary models because they were not captured in a uniform structured format across the entire 15-year period. Incorporating them would have required excluding a substantial number of patients or relying on inconsistent abstraction, thereby risking selection bias and misclassification. Consistent with this rationale, we prespecified covariates based on clinical relevance and data completeness (e.g., age, Hunt-Hess grade, TTM, operation, and key laboratory indices) and used a Fine–Gray framework for GID occurrence to address the competing risk of in-hospital death. Notably, GID was not independently associated with in-hospital mortality after adjustment, which may reflect residual confounding and the absence of GID severity grading in our cohort. Finally, given the observational nature of this analysis, a causal inference between GID and mortality cannot be established. Future multicenter, prospective studies are warranted to validate these findings, explore underlying mechanisms, such as brain-gut-microbiota interactions, and evaluate targeted supportive gastrointestinal interventions in patients with aSAH.

## Conclusion

Gastrointestinal dysfunction was highly prevalent in aSAH and was associated with greater neurological severity, systemic inflammation, and higher resource use. After multivariable adjustment, GID was not independently associated with in-hospital mortality but delineated a high-risk clinical phenotype. These findings support early recognition and structured GI support and highlight the need for standardized severity grading and prospective multicenter evaluation of targeted interventions.

## Data Availability

The original contributions presented in the study are included in the article/supplementary material, further inquiries can be directed to the corresponding author.

## Glossary

ALB: albumin
ALT: alanine aminotransferase
ANS: autonomic nervous system
APTT: activated partial thromboplastin time
aSAH: aneurysmal subarachnoid hemorrhage
AST: aspartate aminotransferase
BMI: body mass index
BUN: blood urea nitrogen
CHD: coronary heart disease
CK: creatine kinase
CKD: chronic kidney disease
CK-MB: creatine kinase-MB
Cr: creatinine
CRP: C-reactive protein
DBIL: direct bilirubin
DBP: diastolic blood pressure
EN: enteral nutrition
GIB: gastrointestinal bleeding
GID: gastrointestinal dysfunction
GOS: Glasgow Outcome Scale
GRV: gastric residual volume
Hgb: hemoglobin
HH score: Hunt-Hess score
HR: heart rate
IL-6: interleukin-6
Lym: lymphocyte count
Myo: myoglobin
NJT: nasojejunal tube
NT-proBNP: N-terminal pro-B-type natriuretic peptide
PCT: procalcitonin
PLT: platelet count
PT: prothrombin time
RBC: red blood cell count
RR: respiratory rate
SBP: systolic blood pressure
sHR: subdistribution hazard ratio
TBIL: total bilirubin
TnT: troponin T
TP: total protein
TTM: target temperature management
WBC: white blood cell count.
